# An early granulocyte colony-stimulating factor treatment attenuates neuropathic pain through activation of mu opioid receptors on the injured nerve

**DOI:** 10.1038/srep25490

**Published:** 2016-05-16

**Authors:** Ming-Feng Liao, Shin-Rung Yeh, Ai-Lun Lo, Po-Kuan Chao, Yun-Lin Lee, Yu-Hui Hung, Kwok-Tung Lu, Long-Sun Ro

**Affiliations:** 1 Department of Life Science, National Taiwan Normal University, 88, Ting-chou Rd., Sec. 4, Taipei, Taiwan; 2 Department of Neurology, Chang Gung Memorial Hospital and College of Medicine, Chang Gung University, 199, Tung Hwa North Rd., Taipei, Taiwan; 3 College of Life Science, National Tsing Hua University, Hsinchu, Taiwan; 4 Institute of Biotechnology and Pharmaceutical Research, National Health Research Institutes, Miaoli, Taiwan

## Abstract

Several studies have shown that the mu opioid receptor (MOR) located in the peripheral nerves can be activated after nerve injury and that it attenuates peripheral nociceptive signals to the spinal dorsal horn. Various cytokines and phosphorylated-p38 (p-p38) activation in the dorsal horn also play an important role in neuropathic pain development. Granulocyte-colony stimulating factor (GCSF) is a growth factor that can stimulate granulocyte formation and has been shown to exert an analgesic effect on neuropathic pain through recruiting opioid-containing leukocytes to the injured nerve. However, the underlying mechanisms are not well understood. Herein, the results of behavior tests in addition to MOR levels in the injured sciatic nerve and the levels of p-p38 and various cytokines in the spinal dorsal horn were studied in vehicle-treated or GCSF-treated chronic constriction injured (CCI) rats at different time points (i.e., 1, 3, and 7 days, respectively) after nerve injury. The results showed that a single early systemic GCSF treatment after nerve injury can up-regulate MORs in the injured nerve, which can decrease peripheral nociceptive signals. Thereafter, those changes suppress the pro-inflammatory cytokine IL-6 but enhance the anti-inflammatory cytokine IL-4, followed by decreases in p-p38 in the dorsal horn, and thus further attenuate neuropathic pain.

Both the aversive sensation of pain following tissue damage^1^ and the development of chronic pain are related to the activation of pro-inflammatory cytokines and microglia in the spinal cord^2^. Previous studies have shown that opioid-containing leukocytes can extravasate to the peripheral tissue after tissue injury or inflammation and there secrete opioids to modulate pain^3^,^4^. Granulocyte-Colony Stimulating Factor (GCSF) is a growth factor that can encourage bone marrow to produce more granulocytes^5^. Our previous studies have shown that early systemic GCSF treatment after peripheral nerve injury can recruit more opioid-containing polymorphonuclear (PMN) cells to the injured site, which can thereafter decrease pro-inflammatory cytokines (IL-1β and TNF-α) in the dorsal root ganglion (DRG) and suppress microglia activation in the dorsal horn^6^. Recently, similar animal studies showed that delayed multiple GCSF treatments can attenuate neuropathic pain after chronic constriction injury (CCI) and spinal cord injury^7^,^8^. Furthermore, human phase I and IIa clinical trials have demonstrated that systemic GCSF treatment can significantly alleviate neuropathic pain in patients with compression myelopathy^9^. Although CGSF has been shown in both human and animal studies to have an analgesic effect on neuropathic pain after nerve injury and compression myelopathy^6^,^7^,^8^,^9^, the underlying mechanisms of the analgesic effects of GCSF on neuropathic pain remain unclear.

Previous studies have shown that phosphorylated-p38 (p-p38) activation in the dorsal horn plays an important role in the development of neuropathic pain^10^,^11^,^12^,^13^. Ji *et al*. found that p-p38 was activated in the spinal cord (restricted entirely to the microglia) soon after (<1 day) spinal nerve ligation (SNL) injury and persisted for >3 weeks^10^. Tsuda *et al*. found similarly that the p-p38 level (exclusively in the microglia) was increased in the dorsal horn at 7 days and 14 days after SNL injury^11^. The suppression of p-p38 by different chemicals can attenuate neuropathic pain development^11^,^12^,^13^. In the microglia, p-p38 interacts with different cytokines to regulate neuropathic pain^13^,^14^,^15^. In addition, the cytokine itself in the spinal cord is also essential for chronic pain development. The pro-inflammatory cytokines IL-1β, IL-6, and TNF-α have been shown to be elevated in the dorsal horn after peripheral nerve injury^16^,^17^. In contrast, the intrathecal injection of an antibody against cytokine IL-6 may attenuate neuropathic pain^18^,^19^. The anti-inflammatory cytokines IL-4 and IL-10 have also been shown to attenuate hyperalgesia^20^,^21^,^22^. GCSF itself is a type of cytokine that can decrease the levels of the pro-inflammatory cytokines TNF-α and IL-1β and increase survival in animals with LPS-induced septic shock^23^. In animal studies, systemic GCSF treatment may suppress the expression of the pro-inflammatory cytokines IL-1β and TNF-α at both the mRNA and protein levels after spinal cord injury^24^,^25^. Delayed systemic GCSF treatments in rats that had received CCI surgery and rats with spinal cord injury can also decrease p-p38 and IL-1β levels in the dorsal horn and suppress neuropathic pain^7^,^8^.

In this study, we examined the underlying mechanisms of the analgesic effects of GCSF on neuropathic pain in CCI rats after a single early systemic GCSF intravenous injection. This study aimed to investigate the temporal changes of mu opioid receptor (MOR) in the injured sciatic nerve and the levels of various cytokines (IL-1β, IL-4, IL-6, TNF-α) and p-p38 in the dorsal horn in CCI rats who received or did not receive GCSF treatment.

## Results

### A single, early systemic GCSF treatment alleviates mechanical allodynia in rats with CCI from the 1st to the 7th day after nerve injury

**Figure 1:**
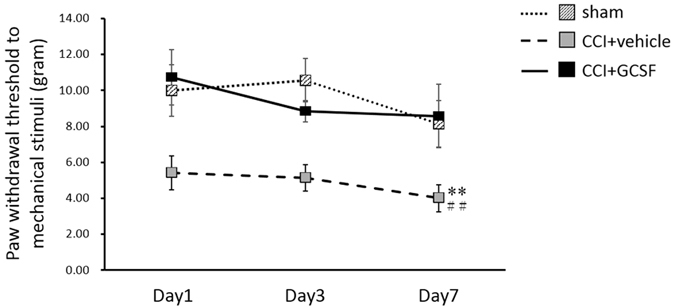
GCSF treatment alleviates mechanical allodynia in rats with CCI. Statistically significant mechanical allodynia developed in the vehicle-treated CCI rats, compared to the sham-operated controls from the 1^st^ to the 7^th^ day after nerve injury (*P* < 0.01). In contrast, early GCSF treatment alleviated mechanical allodynia from the 1^st^ to the 7^th^ day after nerve injury compared to the vehicle-treated CCI rats (*P* < 0.01). The data are shown as the mean ± SEM. Two-way repeated measures ANOVA followed by *post hoc* LSD test. ^##^*P* < 0.01: vehicle-treated groups compared to sham-operated controls. **P < 0.01: GCSF-treated CCI groups compared to vehicle-treated CCI groups *(n* = 7, in each group).

The paw withdrawal thresholds determined by the von Frey filament test (Fig. 1; from the 1^st^ to the 7^th^ day) decreased significantly in the vehicle-treated CCI rats compared to the sham controls from the 1^st^ to the 7^th^ day after nerve injury (Two-way repeated measures ANOVA followed by *post hoc* Fisher’s Least Significant Difference [LSD] test, *P* < 0.01). In contrast, the GCSF-treated CCI rats exhibited significantly attenuated mechanical allodynia compared to the vehicle-treated CCI rats from the 1^st^ to the 7^th^ day after nerve injury (Two-way repeated measures ANOVA followed by *post hoc* LSD test, *P* < 0.01).

### GCSF increased mu opioid receptor (MOR) levels in the injured sciatic nerve from the 1st to the 3rd day after nerve injury

**Figure 2:**
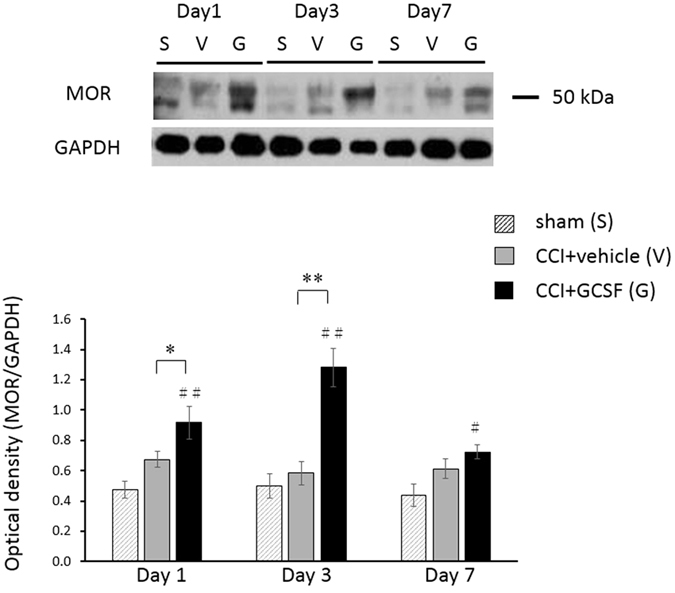
GCSF increased mu opioid receptor (MOR) levels in the injured sciatic nerve. The MOR levels in the injured sciatic nerve, as determined by western blot analysis, were significantly higher in the GCSF-treated CCI rats than in the vehicle-treated CCI rats from the 1^st^ to the 3^rd^ day after nerve injury (*P* < 0.05). The data are shown as the mean ± SEM. ^#^*P* < 0.05, ^##^*P* < 0.01: GCSF-treated groups compared to sham-operated controls. *P < 0.05, **P < 0.01: GCSF-treated CCI groups compared to vehicle-treated CCI groups. One-way ANOVA, *post hoc* LSD test or Kruskal–Wallis, *post hoc* Mann–Whitney rank-sum test, if appropriate (*n* = 5, in each group).

**Figure 3:**
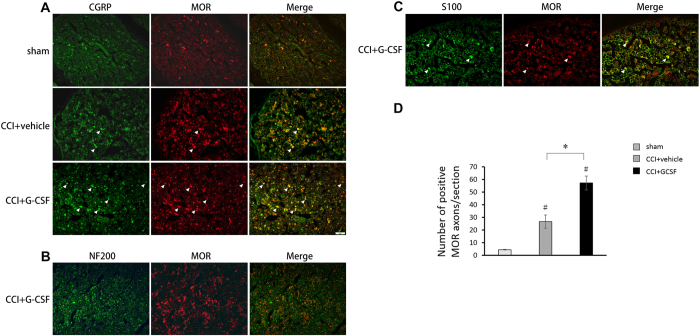
GCSF increased mu opioid receptor (MOR)-positive axons in the injured sciatic nerve. Representative images of double immunofluorescence for MOR (red) and calcitonin gene related peptide (CGRP), a marker of peptidergic small to medium size neurons (green; (**A**)); NF200, heavy subunit of neuro-filament (green; (**B**)); and S100, β-subunit of Schwann cell (green; (**C**)) in the injured sciatic nerve of the sham controls, vehicle-treated CCI rats, and GCSF-treated CCI rats on the 3rd day after nerve injury. (**D**) Quantification of MOR-positive axons in the injured sciatic nerve of sham controls, vehicle-treated CCI, and GCSF-treated CCI rats. A significantly higher number of MOR-positive axons were observed in GCSF-treated CCI rats than in vehicle-treated CCI rats (*P* < 0.05). The MOR-positive axons in the injured sciatic nerve were also slightly higher in vehicle-treated CCI rats than in the sham control rats (*P* < 0.05). Most MORs were co-stained with CGRP-positive axons and S100-positive Schwann cells. There were only a few MOR-positive stained cells co-localized with N200-positive axons. Scale bars = 20 μm. The data are shown as the mean ± SEM. ^#^*P* < 0.05, ^##^*P* < 0.01: GCSF-treated or vehicle-treated groups compared to sham-operated controls. *P < 0.05, **P < 0.01: GCSF-treated CCI groups compared to vehicle-treated CCI groups. Kruskal–Wallis, *post hoc* Mann–Whitney rank-sum test (*n* = 4, in each group). Arrowheads indicate CGRP-positive/MOR-positive axons. Some S100-positive/MOR-positive cells were also observed.

MOR levels in the injured sciatic nerve, as determined by western blot analysis (Fig. 2), were significantly higher in the GCSF-treated CCI rats than in the sham control and vehicle-treated CCI rats from the 1^st^ to the 3^rd^ day after nerve injury (one-way ANOVA, *post hoc* LSD test or Kruskal–Wallis, *post hoc* Mann–Whitney rank-sum test, if appropriate, *P* < 0.05). However, the MOR levels in the injured sciatic nerve were similar for the vehicle-treated CCI rats and GCSF-treated CCI rats on the 7^th^ day after nerve injury. Immunohistochemical studies also revealed a greater number of MOR-positive axons (Fig. 3A–C) in the injured sciatic nerve of GCSF-treated rats than of sham-injured and vehicle-treated rats on the 3^rd^ day after nerve injury (Kruskal–Wallis, *post hoc* Mann–Whitney rank-sum test, *P* < 0.05). The MOR-positive axons in the injured sciatic nerve were higher in vehicle-treated CCI rats than in the sham control rats (Kruskal–Wallis, *post hoc* Mann–Whitney rank-sum test, *P* < 0.05). Double immunofluorescence studies showed that most MORs were mainly co-stained with CGRP (a marker of peptidergic small to medium sized neurons)-positive axons and S100 (β-subunit of Schwann cell)-positive Schwann cells. However, only a few MORs were co-localized with N200-positive axons.

### GCSF decreased the pro-inflammatory cytokine IL-6 but increased the anti-inflammatory cytokine IL-4 from the 1st to the 7th day after nerve injury

**Figure 4:**
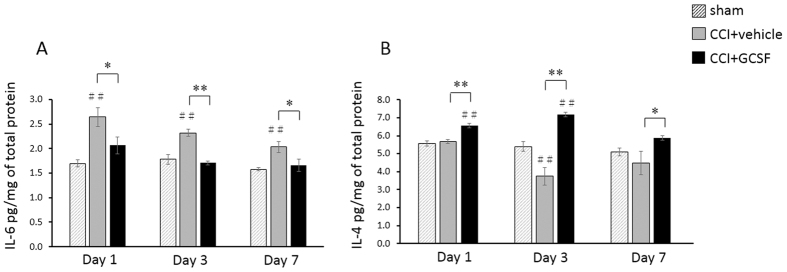
GCSF decreased the pro-inflammatory cytokine IL-6 but increased the anti-inflammatory cytokine IL-4. (**A**) ELISA study revealed significantly higher pro-inflammatory cytokine IL-6 levels in the dorsal horn of the vehicle-treated CCI rats than in the sham controls (*P* < 0.01) from the 1^st^ to the 7^th^ day after nerve injury. In contrast, significantly lower IL-6 levels were observed in the dorsal horn of GCSF-treated CCI rats than in the vehicle-treated CCI rats from the 1^st^ to the 7^th^ day after nerve injury (*P* < 0.05). (**B**) ELISA study revealed significantly lower anti-inflammatory cytokine IL-4 levels in the dorsal horn of the vehicle-treated CCI rats than in the sham controls on the 3^rd^ day after nerve injury (*P* < 0.01). In contrast, significantly higher IL-4 levels were observed in the dorsal horn of GCSF-treated CCI rats than in the vehicle-treated CCI rats from the 1^st^ to the 7^th^ day after nerve injury (*P* < 0.05). The data are shown as the mean ± SEM. ^#^*P* < 0.05, ^##^*P* < 0.01: GCSF-treated or vehicle-treated groups compared to sham-operated controls. *P < 0.05, **P < 0.01: GCSF-treated CCI groups compared to vehicle-treated CCI groups. One-way ANOVA, *post hoc* LSD test (*n* = 5, in each group).

The ELISA study of different cytokine levels in the spinal dorsal horn revealed that pro-inflammatory cytokine IL-6 levels (Fig. 4A) were significantly higher in the vehicle-treated CCI rats than the sham controls from the 1^st^ to the 7^th^ day after nerve injury (one-way ANOVA, *post hoc* LSD test, *P* < 0.01). In contrast, anti-inflammatory cytokine IL-4 levels (Fig. 4B) in the spinal dorsal horn were decreased in vehicle-treated CCI rats compared to the sham controls only on the 3^rd^ day after nerve injury (one-way ANOVA, *post hoc* LSD test, *P* < 0.01). The GCSF-treated CCI rats exhibited significantly lower pro-inflammatory cytokine IL-6 levels (one-way ANOVA, *post hoc* LSD test*, P* < 0.05) than the vehicle-treated CCI rats but significantly higher anti-inflammatory cytokine IL-4 levels from the 1^st^ to the 7^th^ day after nerve injury (one-way ANOVA, *post hoc* LSD test, *P* < 0.05). However, there were no significant differences in the levels of other cytokines, including IL-1β and TNF-α, in the dorsal horn among the sham controls, vehicle-treated, and GCSF-treated CCI rats (data not shown).

### GCSF decreased the p-p38/total p38 ratio in the spinal dorsal horn on the 3rd day after nerve injury

**Figure 5:**
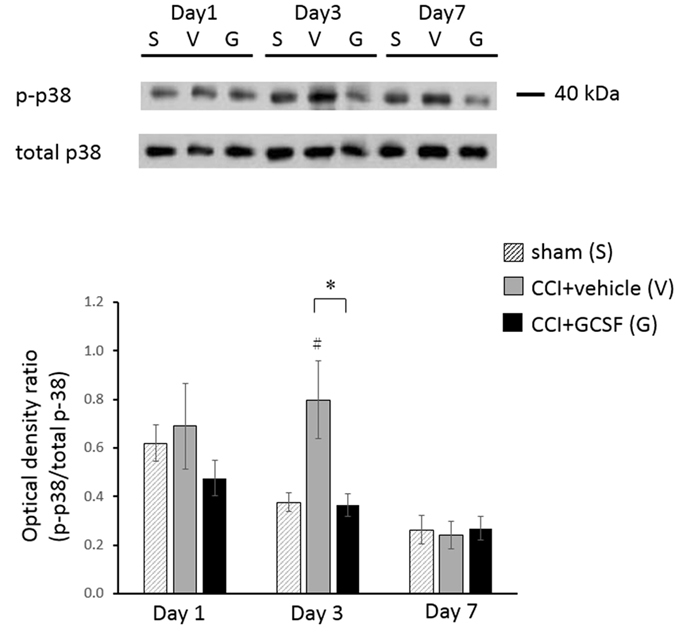
GCSF decreased the p-p38/total p38 ratio in the spinal dorsal horn. The phosphorylated-p38/total p38 ratio in the spinal dorsal horn, as determined by western blot analysis, was significantly higher in vehicle-treated CCI rats than in sham controls on the 3^rd^ day after nerve injury (*P* < 0.01). In contrast, a significantly lower p-p38/total p38 ratio was observed in the dorsal horn of GCSF-treated CCI rats than in vehicle-treated CCI rats on the 3^rd^ day after nerve injury (*P* < 0.01). The data are shown as the mean ± SEM. ^#^*P* < 0.05, ^##^*P* < 0.01: vehicle-treated groups compared to sham-operated controls. *P < 0.05, **P < 0.01: GCSF-treated CCI groups compared to vehicle-treated CCI groups. One-way ANOVA, *post hoc* LSD test or Kruskal–Wallis, *post hoc* Mann–Whitney rank-sum test, if appropriate (*n* = 6, in each group).

**Figure 6:**
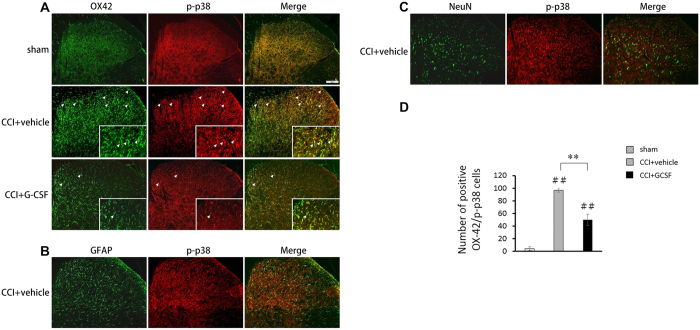
GCSF decreased p-p38-positive cells in the dorsal horn. Representative images of double immunofluorescence in the dorsal horn for p-p38 (red) and OX-42, a microglia marker (green; (**A**)); GFAP, an astrocyte marker (green; (**B**)); and NeuN, a neuronal marker (green; (**C**)) of the sham controls, vehicle-treated CCI rats, and GCSF-treated CCI rats on the 3^rd^ day after nerve injury. Most p-p38 was co-stained with OX-42-positive cells. (**D**) Quantification of the OX-42/p-p38-positive cells in the right quarter part of the spinal dorsal horn (lamina I–V) of the sham controls, vehicle-treated CCI, and GCSF-treated CCI rats. The vehicle-treated CCI rats had a significantly higher number of OX-42/p-p38-positive cells than the sham control rats on the 3^rd^ day after nerve injury (*P* < 0.01); in contrast, the GCSF-treated CCI rats had a significantly lower number of OX-42/p-p38-positive cells than the vehicle-treated CCI rats on the 3^rd^ day after nerve injury (*P* < 0.01). Scale bars = 20 μm. The data are shown as the mean ± SEM. ^#^*P* < 0.05, ^##^*P* < 0.01: GCSF-treated or vehicle-treated groups compared to sham-operated controls. *P < 0.05, **P < 0.01: GCSF-treated CCI groups compared to vehicle-treated CCI groups. Kruskal–Wallis, *post hoc* Mann–Whitney rank-sum test (*n* = 5, in each group). Arrowheads indicate OX-42-positive/p-p38-positive cells.

The p-p38/total p38 ratios (Fig. 5) examined by western blot analysis in the spinal dorsal horn of the vehicle-treated CCI rats were significantly higher than in the sham controls on the 3^rd^ day after nerve injury (Kruskal–Wallis, *post hoc* Mann–Whitney rank-sum test, *P* < 0.05). In contrast, these increased p-p38 protein levels were suppressed significantly by GCSF treatment only on the 3^rd^ day after nerve injury (Kruskal–Wallis, *post hoc* Mann–Whitney rank-sum test, *P* < 0.05). However, the p-p38 protein levels in the spinal dorsal horn were similar among sham-operated rats, vehicle-treated CCI rats, and GCSF-treated CCI rats on the 1^st^ day and 7^th^ day after nerve injury. In the immunohistochemical experiments, most p-p38-positive cells in the dorsal horn were co-stained with OX-42-positive cells (microglia) (Fig. 6A–C). There was a significantly higher number of OX-42/p-p38-positive cells in the right quarter part of the spinal dorsal horn (lamina I–V) in the vehicle-treated CCI rats than in the sham-operated controls (Kruskal–Wallis, *post hoc* Mann–Whitney rank-sum test, *P* < 0.01) on the 3^rd^ day after nerve injury; in contrast, the GCSF-treated CCI rats had a lower number of OX-42/p-p38-positive cells than the vehicle-treated CCI rats (Kruskal–Wallis, *post hoc* Mann–Whitney rank-sum test, *P* < 0.01) on the 3^rd^ day after nerve injury (Fig. 6D).

## Discussion

Our studies showed that a single early systemic GCSF treatment can suppress neuropathic pain^6^. Carvalho and Liou’s studies showed opposite findings in that GCSF treatments could induce mechanical hyperalgesia by activating spinal mitogen-activated protein (MAP) kinases^26^ and increasing the pro-inflammatory cytokine levels in the injured nerve^27^. However, they used different GCSF doses and administration routes, and they tested pain behaviors at different, early time points. In Carvalho’s study, the intraplantar injection of GCSF induced mechanical hyperalgesia that was detected only at 1–7 hours after GCSF (100–200 ng) administration, and high-dose GCSF (300 ng) induced significant hyperalgesia from 1–24 h after GCSF administration^26^. Our previous studies showed that systemic GCSF injection can suppress neuropathic pain approximately 1 day after administration and achieve its maximal effect on the 3^rd^ day after nerve injury^6^. That finding may explain why Carvalho could not detect the analgesic effect of GCSF on neuropathic pain: because this effect of GCSF can only be detected 24 h after nerve injury. In Liou’s study, the mechanical hyperalgesia persisted from the 1^st^ to the 14^th^ day after sciatic nerve ligation. However, GCSF (0.25 mg/kg) was given subcutaneously daily beginning 5 days before sciatic nerve ligation^27^. The routes, doses, and time points of GCSF administration were quite different from ours, which may explain why there was a discrepancy between our study and Liou’s. One possible reason is that continuous subcutaneous GCSF injections may not recruit enough opioid containing PMN cells to the injured nerve to alleviate neuropathic pain. In contrast, similar recent studies showed that delayed continuous systemic GCSF treatments (15 μg/kg for 5 consecutive days, 2 to 3 weeks after nerve injury) could also attenuate mechanical allodynia in both CCI^7^ and spinal cord injury^8^ models by inhibiting the pro-inflammatory cytokine IL-1β in the dorsal horn. These results are consistent with our studies. Moreover, similar analgesic effects of repeated systemic GCSF treatments were also observed in patients with compression myelopathy in human phase I and IIa clinical trials^9^.

In addition to relieving neuropathic pain, GCSF also exhibited other neuro-protective effects in many studies with different animal models. Repeated systemic GCSF treatments over 5 days after spinal cord injury can promote hind-limb functional recovery in rats by suppressing the mRNA expression of the pro-inflammatory cytokines IL-1β and TNF-α in the spinal cord^24^. Subcutaneous GCSF treatment for 3 consecutive days after hemisection of the mouse spinal cord can decrease the mRNA expression levels of pro-inflammatory cytokines and increase the mRNA levels of anti-inflammatory cytokines in the microglia in the spinal cord^25^. Based on both animal models and human studies, we believe that systemic GCSF treatment(s) can exert anti-inflammatory and analgesic effects, resulting in the attenuation of neuropathic pain and protection of the nervous system.

In addition to acting on the CNS, opioids also act on the MORs in the peripheral nerves and suppress pain^28^. In the peripheral nerves, MORs are mainly expressed in small- and medium-diameter dorsal root ganglion (DRG) neurons or axons and are co-expressed with substance P (SP) and calcitonin gene-related (CGRP)^29^,^30^,^31^. In CCI injured rats, increased MOR levels were found in the injured sciatic nerve, especially in the proximal part, at both 2 and 14 days after nerve injury^30^,^31^. Moreover, local morphine and encephalin (a MOR agonist) can attenuate mechanical hyperalgesia, suggesting that these MORs in the peripheral nerve are functional^31^. After tissue injury or inflammation, opioid-containing leukocytes can extravasate to the peripheral tissue and secrete endogenous opioids, which can reverse mechanical or thermal hyperalgesia^3^,^4^,^32^,^33^. The opioid receptors are synthesized in the dorsal root ganglion, up-regulated and transported to the injured peripheral nerve, further increasing the density of opioid receptors in the injured nerve and suppressing peripheral nociceptive signal transduction^3^,^4^,^34^,^35^,^36^. Similarly to earlier studies, our recent study had shown that a single, early GCSF treatment can probably enhance endogenous opioid secretion by recruiting more opioid-containing PMN cells to the injured tissue as early as 12 hours after nerve injury, and this analgesic effect can be blocked by naloxone methiodide (NLXM) injection^6^. In this study, we found the MOR levels in the injured sciatic nerve to be significantly elevated after GCSF treatment from the 1^st^ to the 3^rd^ day after CCI nerve injury, and those MORs were mainly co-stained with CGRP-positive axons and S100-positive Schwann cells. Given the time sequence and the effect of naloxone injection, the increased endogenous opioid synthesis following GCSF treatment could act on MORs at the injured nerve, up-regulate MOR synthesis and transport, and thereafter possibly suppress peripheral nociceptive signal transduction, resulting in the alleviation of neuropathic pain development. However, there have been no similar studies reporting the function of the MORs in Schwann cells. Thus, the role of the MORs in Schwann cells remains uncertain and warrants further investigation.

Peripheral opioid receptors in the dorsal root ganglion (DRG) are also coupled to G proteins^37^. The up-regulation of opioid receptors in the peripheral nerves may inhibit the nerve action potentials produced by nociceptive stimuli in the peripheral nerves^38^,^39^. Whole cell patch clamp studies showed that the MOR agonist inhibited both high-threshold calcium current and TTX-resistant Na current in the DRG neuron^40^,^41^. Electrophysiological study of the spinal cord slice showed that the binding of opioids to MORs can reduce nociceptive signal transmission at the central Aδ- and C-fiber synapses by the inhibition of presynaptic voltage-dependent Ca channels (VDCC)^35^. Our previous study also showed that the peripheral opioid receptor antagonist naloxone methiodide (NLXM) can reverse the analgesic effects of GCSF, and those findings further confirmed that the analgesic effects of GCSF are produced through the opioid/opioid receptor pathway in the injured nerve^6^. When the peripheral afferent nerve received nociceptive stimulation, various cytokines, including IL-1β, TNF-α, and IL-6, were released from central terminals in the spinal dorsal horn^42^. Those cytokines contributed to central sensitization in the spinal cord and thus enhanced neuropathic pain development^43^. In our study, pro-inflammatory cytokine IL-6 levels increased and anti-inflammatory cytokine IL-4 levels decreased on the 1^st^ and 3^rd^ days after nerve injury, respectively. Those changes in cytokines were reversed after GCSF administration. Pre-treatment with i.v. GCSF can increase the serum levels of the pro-inflammatory cytokines TNF-α and IL-6 in healthy volunteers after endotoxin challenge. However, those effects were only observed in the first few hours after GCSF administration^44^,^45^. The direct effect of GCSF may be due to the enhanced production of pro-inflammatory cytokines. In contrast to the short-term direct effect, the indirect long-lasting and anti-inflammatory effects of GCSF are more complex and are probably related to the modulation of complex cytokine networks. Based on the temporal sequence observed in our studies, the effect of systemic GCSF treatment on neuropathic pain is not through the direct activation of cytokine levels on the dorsal horn. Instead, we propose that GCSF recruits more opioid-containing PMN cells to secrete opioids and up-regulates the MORs in the injured nerve. This process is followed by decreased nociceptive signals to the spinal cord and decreased pro-inflammatory cytokine IL-6 as well as enhanced anti-inflammatory cytokine IL-4 in the spinal dorsal horn, suppressing neuropathic pain.

Many studies using different animal models have shown that p-p38 activation in microglia in the spinal dorsal horn plays an important role in the development of neuropathic pain^10^,^11^,^12^,^13^. Our studies produced a similar result to the work of Xu *et al*.^13^, which showed the p-p38 level on the dorsal horn to be elevated at the 3^rd^ day after CCI surgery. In the microglia, p-p38 may interact with different cytokines to modulate neuropathic pain^13^,^14^,^15^. For example, IL-6 may increase p-p38 in the microglia after CCI surgery^19^. Our study showed a similar result in that IL-6 increased in the spinal dorsal horn, preceding the increase in p-p38 after nerve injury, and these changes were reversed by systemic GCSF treatment. These findings suggested that the decreased levels of activated pro-inflammatory cytokines probably inhibit p-p38 activation in the dorsal horn and further attenuate neuropathic pain. Multiple delayed systemic GCSF treatments in rats with CCI and spinal cord injury could also decrease p-p38 levels in the dorsal horn and suppress neuropathic pain^7^,^8^. In addition to its suppression of neuropathic pain in animal models, intrathecal GCSF injection can also suppress the increase in p-p38 and reduce motor function defects in rats with spinal cord ischemia^46^. In contrast, *ex vivo* studies showed that the exposure of human umbilical vein endothelial cells to GCSF resulted in increased p-p38. The effects began several minutes after GCSF exposure and declined 30 minutes later^47^. Another similar cell culture study showed that the p-p38 level increased only from 5 to 120 minutes after GCSF stimulation^48^. Furthermore, GCSF has a small molecular weight (19.6 kDa) and is able to cross the intact blood brain barrier (BBB)^49^. GCSF also has a short half-life (4 hours in rodents)^50^, which suggests that the direct effect of GCSF may only persist for a short period of time. Based on all these studies, we have concluded that p-p38 can be activated *ex vivo* soon after direct GCSF exposure. However, GCSF also has indirect effects that suppress p-p38 activation in the dorsal horn after nerve injury to attenuate neuropathic pain and preserve nerve function in various *in vivo* animal studies^10^,^11^,^12^,^13^,^46^, and these effects were probably through the modulation of various cytokines. It is also consistent with the temporal sequence that GCSF attenuated neuropathic pain from 24 hours and persisted following nerve injury for at least 25 days^6^.

**Figure 7:**
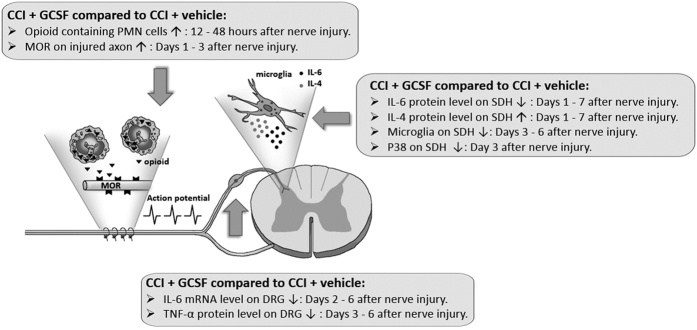
The time sequence of the effects of G-CSF on MOR levels in the injured nerve and of the cytokine levels and microglial/p-p38 activation in the spinal dorsal horn. Compared to vehicle-treated CCI rats, the sequential effects of GCSF on GCSF-treated CCI rats increased opioid containing polymorphonuclear (PMN) cells from 12 to 48 hours and increased mu opioid receptor (MOR) levels on days 1–3 at the nerve ligature site after nerve injury; decreased IL-6 and TNF-α levels on days 2–6, and days 3–6, respectively, in the dorsal root ganglion (DRG) after nerve injury; decreased IL-6 but increased IL-4 levels from days 1–7 in the spinal dorsal horn (SDH) after nerve injury; and suppressed microglia and p-p38 activation on days 3–6 and on day 3, respectively, in the spinal dorsal horn after nerve injury. (▼: Opioid, 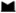: Mu opioid receptor [MOR], 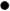: IL-6, 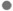: IL-4, ↑: increase, ↓: decrease.)

Our studies demonstrated that a single, early systemic GCSF treatment may attenuate neuropathic pain through the endogenous opioid/opioid receptor pathway in the injured nerve. The systemic GCSF treatment can recruit far more opioid-containing PMNs to the injured nerve. Thereafter, the endogenous opioids up-regulate the MOR levels of the injured sciatic nerve, which can reduce nociceptive signal transmission at central Aδ- and C-fiber synapses to the spinal cord dorsal horn^35^. The pro-inflammatory cytokine IL-6 was down-regulated, but the anti-inflammatory cytokine IL-4 was up-regulated in the spinal dorsal horn through the decreased nociceptive signals. Then, the p-p38 level in the dorsal horn was probably further suppressed by those cytokine changes, all of which can attenuate neuropathic pain development (Fig. 7).

The endogenous opioid secretion produced by GCSF administration may avoid the addiction side effect induced by repeated exogenous opioid treatment. Moreover, several studies have shown that both a single, early systemic treatment and multiple, delayed GCSF treatments after nerve injury can attenuate neuropathic pain^7^,^8^. Thus, single early and multiple delayed systemic GCSF treatments provide a new therapeutic strategy with excellent potential to treat neuropathic pain. However, the effects of systemic GCSF treatments on neuropathic pain are greatly dependent on the time of administration, dosage and administration route, which all require further investigation.

## Methods

### Subjects

Adult male Sprague-Dawley rats (BioLASCO Taiwan Co., Ltd., Taipei, Taiwan) weighing approximately 300–350 g were used. The animal room was artificially lit from 6:00 h to 18:00 h. Three rats were housed in a temperature-controlled (22 °C) cage; water and pellets of rat food were available *ad libitum*. All behavioral tests were performed during the light cycle. All procedures were conducted in accordance with the guidelines of the International Association for the Study of Pain (IASP)^51^ and were approved by the Institutional Animal Care and Use Committee (IACUC) at the Chang Gung Memorial Hospital (No: 2013050901). Every procedure was made to minimize animal suffering as well as the number of animals required to generate reliable experimental data.

### Surgical procedure and protocols of drug treatment

Rats were deeply anesthetized with sodium pentobarbital (50 mg/kg, intraperitoneal (i.p.) injection before operation) (SCI Pharmtech Inc., Taiwan) and then received chronic constriction injury (CCI) surgery according to the Bennett’s model^52^. Four ligatures of 4–0 chromic gut were loosely tied around the proximal part of the right sciatic nerve with approximately 1.0–1.5 mm intervals between the knots. At the time of tying, the ligatures just barely reduced the nerve diameter, and the epineural circulation was preserved. A separate group of rats underwent sham operations that only involved the dissection of muscle without manipulation of the right sciatic nerve. A single dose of GCSF (200 μg/kg, Filgrastim; Kyowa Hakko Kirin, Japan) was injected intravenously (i.v.) immediately after surgery. The same amount of normal saline was injected in the vehicle control groups.

### Behavioral tests for mechanical allodynia

To avoid bias, surgery and behavioral testing were performed by 2 different investigators. Rats were investigated by observers blinded to the treatment of the animals. Prior to testing, each animal was placed in a transparent 30 × 30 × 15 cm^3^ box for a 10 min habituation period. No food or water was available to the rats during the experiment. Mechanical allodynia was performed by von Frey hair, according to a previously described protocol^53^. Von Frey hairs were applied to the central region of the right plantar surface of a hind paw in ascending order of force (0.6, 1.0, 1.4, 2.0, 4, 6, 8, 10, 15, and 26 g). When the rats showed a sharp withdrawal response or an immediate flinch to a given filament, the bending force of that filament was defined as the mechanical threshold intensity. The hind paw withdrawal threshold was defined as the lowest force that caused at least three withdrawals out of five consecutive applications^53^. The experimental conditions were identical for the sham-operated and experimental rats.

### Western blotting assay and enzyme-linked immunosorbent assay (ELISA)

We used a western blotting assay to check the MOR levels in the injured nerve (at the site of the four chromic gut ligatures and in the same region of the sham rats) and the p-p38 level in the dorsal horn (the right quarter part of the spinal cord), and we used an ELISA assay to check various cytokine levels (IL-1β, IL-4, IL-6, TNF-α) in the dorsal horn of sham-injured and experimental rats. Rats were anesthetized with sodium pentobarbital and transcardially perfused with PBS (Sigma, USA). The sciatic nerve at the chronic constriction injury site (at the site with four chromic gut ligatures) and the right quarter part of the L4-L6 segments of the spinal cord were quickly separated and collected in a tissue lysis buffer (T-PER® Tissue Protein Extraction Reagent, Thermo Scientific, USA) and a “cocktail” of protease inhibitors (Roche, Germany) and phosphatase inhibitor (Roche, Germany). After homogenization, the tissue lysates were centrifuged at 13,500 rpm for 45 min at 4 °C. Equal protein loading from the dorsal horn and injured sciatic nerve was verified using Coomassie brilliant blue staining after sodium dodecyl sulfate polyacrylamide gel electrophoresis (SDS-PAGE). Protein samples were separated by SDS-PAGE and transferred onto polyvinylidene fluoride (PVDF) membranes. The blots were blocked using 5% bovine serum albumin (BSA) (Amresco, USA) in Tris-buffered saline (TBS) with 0.1% Tween-20 overnight at 4 °C and then incubated with anti-phospho-p38 (1:1000; Cell Signaling Technology, USA), p38 (1:1000; Cell Signaling Technology, USA), MOR-1 (receptor for β-endorphin; 1:200; Santa Cruz Biotechnology, USA), or GAPDH (internal control; 1:6000; Cell Signaling Technology, USA) primary antibody in TBS containing 0.1% Tween-20 overnight at 4 °C. This step was followed by incubation with horseradish peroxide (HRP)-linked secondary antibody (1:10000, anti-rabbit IgG; Millipore, USA) for 60 min at room temperature. All washing was performed using TBS containing 0.1% Tween-20. The bands were detected using Image-ProPlus 5.0 (Media Cybernetics Inc., Silver Spring, MD). According to the method instructions from Santa Cruz Biotechnology (MOR-1 [H-80]: sc-15310, Santa Cruz Biotechnology, USA), two MOR bands indicated two isoforms with variable extents of N-glycosylation, which are visible in the western blot analysis. These two bands must be considered together for quantification, as described in several published studies^54^,^55^,^56^.

In the ELISA studies, the spinal dorsal horn expression of IL-1β, IL-4, IL-6, and TNF-α protein were determined using IL-1β (Thermo Scientific, USA), IL-4 (Novex Life Technology, USA), IL-6 (Invitrogen, USA), and TNF-α (Invitrogen, USA), respectively. Samples (50 μΛ of dorsal horn lysate containing 10 μg of total tissue protein) were analyzed in triplicate following the manufacturer’s instructions. The microplates were read by means of the absorbance at the 450 nm wavelength in an ELISA plate reader (SpectraMax M5, Molecular Devices Corporation Sunnyvale, California).

### Immunohistochemistry

Rats were deeply anesthetized with sodium pentobarbital and transcardially perfused with PBS (Sigma, USA), followed by a fixative solution containing 4% paraformaldehyde. The right quarter of the L4-L6 segments of the spinal cord and injured sciatic nerves (at the site of four ligatures) were resected, placed in 4% paraformaldehyde for 4 h and transferred to 30% sucrose at 4 °C overnight. The samples were subsequently embedded in OCT compound (Tissue-Tek 4583; Sakura, Tokyo, Japan) and rapidly frozen. For immunostaining of the sciatic nerve and spinal dorsal horn, every fourth section was picked from a series of consecutive sciatic nerve (10 μm) and spinal cord sections (15 μm). Tissue sections from the injured sciatic nerve and spinal cord were obtained using a freezing microtome (CM 3050; Leica, Nussloch, Germany) and mounted on polylysine-coated slides.

For double immunofluorescence analysis of the injured sciatic nerves, all of the sections were blocked with protein-blocking buffer (BioGenex, San Ramon, USA) and incubated overnight at 4 °C with primary antibodies: rabbit anti-rat Mu opioid receptor (MOR, 1:500; Abcam, UK) mixed with goat anti-rat calcitonin gene related peptide (CGRP, 1:100, Abcam, UK), mouse anti-rat neurofilament 200 (NF 200, clone N52, 1:500, Sigma, USA) or mouse anti-rat S100 β-subunit (Schwann cell, 1:200; Sigma, USA), followed by the secondary antibodies (FITC-conjugated goat anti-mouse IgG; TRITC-conjugated goat anti-rabbit IgG and FITC-conjugated donkey anti-goat IgG; TRITC-conjugated donkey anti-rabbit IgG, 1:500, Jackson Immuno Research Laboratories, USA). Sections were then incubated for 1 min at room temperature with DAPI (nuclear staining, 4′,6-diamidino-2-phenylindole, 1:10000, Enzo Life Sciences) before being mounted for imaging by fluorescence microscope.

For double immunofluorescence analysis of the spinal dorsal horn, spinal sections were incubated overnight at 4 °C with rabbit anti-rat phospho-p38 (1:100; Cell Signaling Technology, USA), mixed mouse anti-rat NeuN (Neuronal specific nuclear protein, 1:500; Millipore, USA), mouse anti-rat GFAP (Astrocyte marker, 1:500; Millipore, USA), or mouse anti-rat OX-42 (Microglia marker, 1:500; Millipore, USA). Afterward, sections were incubated for 1 h at room temperature with the secondary antibodies (TRITC-conjugated goat anti-rabbit and FITC-conjugated goat anti-mouse, 1:250; Jackson Immuno Research Laboratories, USA). The images were acquired using a fluorescence microscope (Olympus BX51, Japan) connected to a digital camera and computer; montages were created and analyzed using Image-ProPlus 5.0 (Media Cybernetics Inc., Silver Spring, MD). The number of positive p-p38 stained cells in the right quarter of the L4-L6 segments of the spinal cord (lamina I–V) and MOR-positive stained axons in the injured sciatic nerve were counted by a different investigator who was blinded to the animals’ status. Omission of the primary antibody served as the negative control for all experiments and showed no staining.

### Statistical Analysis

Statistical analyses were performed using the Statistical Program for Social Sciences (SPSS) software (version 19.0; IBM, USA). Quantitative data were plotted as the mean ± standard error of mean (SEM). For behavioral experiments, two-way repeated measures ANOVA were conducted, followed by *post hoc* Fisher’s Least Significant Difference (LSD) test to compare the difference between each group. For other multiple comparisons, we used the Shapiro-Wilk test to check whether the data were normally distributed. Normally distributed data were further analyzed by one-way analysis of variance (ANOVA) (for 3 or more groups) followed by a *post hoc* LSD test. If the data lacked normality, the Kruskal–Wallis and *post hoc* Mann–Whitney rank-sum tests (two-tail) were used. The equal variance of data of each group was checked by the Levene homogeneity of variance test before ANOVA test. If the data lacked equal variance, the Games Howell test was used. *P* values less than 0.05 were considered statistically significant.

## Additional Information

**How to cite this article**: Liao, M.-F. *et al*. An early granulocyte colony stimulating factor treatment attenuates neuropathic pain through activation of mu opioid receptors on the injured nerve. *Sci. Rep.* **6**, 25490; doi: 10.1038/srep25490 (2016).
